# Polypyrrole Derivatives: Preparation, Properties and Application

**DOI:** 10.3390/polym16162233

**Published:** 2024-08-06

**Authors:** Lu Hao, Changyi Dong, Demei Yu

**Affiliations:** 1 State Key Laboratory of Electrical Insulation and Power Equipments, MOE Key Laboratory for Non-Equilibrium Synthesis and Modulation of Condensed Matter, School of Chemistry, Xi’an Jiaotong University, No. 28 Xianning West Road, Xi’an 710049, China; xuqihao_fighting@stu.xjtu.edu.cn (L.H.); changyi@stu.xjtu.edu.cn (C.D.); 2 Department of Materials Engineering, Shaanxi Polytechnic Institute, No. 12 Wenhui West Road, Xianyang 712000, China

**Keywords:** PPy derivatives, side substitution, N-site substitution, copolymers

## Abstract

Polypyrrole (PPy) has attracted widespread attention due to its excellent environmental stability, high conductivity, simple synthesis, good biocompatibility, and reversible redox properties. PPy derivatives not only inherit the advantages of polypyrrole, but also have some unique properties. The side and N-site substitution of PPy can not only yield polymers with good solubility, but it also endows polymers with special functionalities by controlling the introduced functional groups. The performance of copolymers can also be adjusted by the type of monomer or polymerization ratio. In this review, an overview of the different types, main preparation methods, and the application prospects of PPy derivatives reported to date are summarized and presented. The current challenges and future opportunities in this research area are also prospected.

## 1. Introduction

Conductive polymers (CPs) are a special type of conjugated polymer with inherent conductivity. Due to their unique polymer properties, excellent electrical and mechanical properties, stability, and biocompatibility, they have broad application prospects in fields such as energy storage [1,2], flexible electronics [3], and bioelectronics [4]. Among numerous conductive polymers, PPy has become a hot research topic due to its high conductivity, good environmental stability, easy synthesis, fast redox conversion, and good biocompatibility. It has high potential application value in various fields such as biochemical sensors, optoelectronic devices, metal anti-corrosion, and biomedicine. However, PPy also has some unsatisfactory aspects, such as poor solubility in organic or inorganic solvents due to strong intermolecular and intramolecular interactions, and poor processability of the material. This greatly limits the potential application of PPy in many fields. So far, the relevant researchers have reported various preparation methods to improve the processability of PPy. Common methods include the following: (1) Preparation of composite materials of PPy and conventional polymers. Conventional polymers typically use materials with good physical and mechanical properties. The composite material prepared in this way can simultaneously exhibit excellent processability and controllable conductivity [5,6]. (2) Preparation of soluble PPy using functional dopants. The dopants used can usually provide larger doped ions for PPy chain, such as amphiphilic macromolecules like dodecylbenzene sulfonic acid [7,8]. Due to the large size of anions, PPy molecular chains can be isolated to a certain extent, reducing inter chain and intra chain interactions, thereby improving the solubility of PPy. (3) Preparation of PPy nano dispersion system using surfactants or stabilizers. (4) Introduction of suitable groups on the pyrrole ring, or the use of chemical or electrochemical methods to prepare copolymers of PPy, and conventional polymers to prepare PPy derivatives. Due to the convenience of polymerization and the high selectivity of introducing groups, and the fact that the properties of copolymer materials can be adjusted by the type or polymerization ratio of the copolymer material, there have been many reports on the research of this method. The conductive polymer prepared using this method not only has good solubility, but also can endow the polymer with special functionality by controlling the introduced groups or copolymerization components.

There have been hundreds of reviews on conductive polymers or PPy between 2014 and 2024, but there is very little content on PPy derivatives, which does not allow readers to systematically understand the efforts made by researchers in the field of PPy derivatives. Furthermore, the search in the Web of Science database showed that just two reviews on this topic were published in the years 2014–2024 [9,10]. Camurlu [9] had mainly discussed the electrochromic application of polypyrrole derivatives. However, there were no more than 10 articles on this research in our article. In addition, this article was published in 2014, most of the references cited were earlier, and there was almost no intersection with the references we cited. Jarosz et al. [10] mainly discussed electrochemically produced copolymers of pyrrole and its derivatives. However, the PPy derivatives discussed in our paper include side-substituted PPy and N-substituted PPy in addition to pyrrole copolymers. The polymerization methods include electrochemical methods and chemical oxidation methods. In addition, this article cited too many references published before 2010, specifically up to 75 (a total of 124 refs).

With this review, we aimed to help more readers understand some of the research progress of PPy derivatives in the last ten years, so as to better promote the further development and application of PPy derivatives. In addition, our research group has conducted a series of works in the field of PPy derivatives. Based on this, we reviewed the main types of PPy derivatives (side-substituted PPy derivatives, N-substituted PPy derivatives and copolymer-based pyrrole), the preparation methods of PPy derivatives (chemical oxidation and electrochemical methods), and the application fields of PPy derivatives in the past decade in this paper. Finally, the current challenges and future opportunities in this research area are prospected.

## 2. The Types of PPy Derivatives

There are three types of PPy derivatives, namely N-substituted PPy, side-substituted PPy, and copolymer-based pyrrole. It has been found that the density, solubility, thermal stability and other properties of PPy derivatives obtained by substitution or copolymerization of pyrrole monomers will be improved.

### 2.1. Side Substitution of PPy

**Figure 1:**
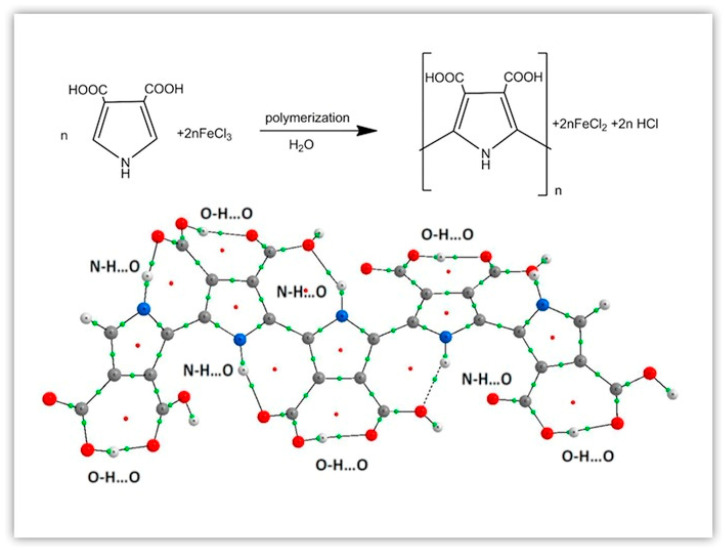
The synthesis of polypyrrole 3,4-dicarboxylic acid [15]. Copyright 2019 Polymer.

Because the polymerization of pyrrole mainly occurs at α-position, the side substitution of pyrrole mainly occurs at β-position. Introducing specific functional groups at β-position of the pyrrole ring can effectively improve the solubility and processability [11], mechanical properties [12,13], thermal stability [14], electrical optics [15,16,17,18], hydrophobicity [19,20], and other properties of PPy. Mizera et al. [15] synthesized a new poly(pyrrole-3,4-dicarboxylic acid) (PPyDCA) (as shown in Figure 1) and studied its optical, electrical and structural properties.

It has been found that the types of side substituents have a significant impact on the structure and properties of polymers. Santos et al. [21] evaluated the influence of the existence of the push–pull electron group on the structure and electronic properties of PPy derivatives through semi empirical and Density functional theory. The results show that the semiconductivity of PPys is maintained after the encapsulation process, that is, the complexation process does not affect the HOMO–LUMO gaps. In addition, solvents also have an impact on the polymerization reaction. Krukiewicz et al. [22] studied the effect of solvents on the synthesis and physicochemical properties of poly(3,4-ethylenedioxypyrrole). It was found that compared to organic solvents such as CH_3_CN and CH_2_Cl_2_, aqueous solutions are more favorable for the polymerization of 3,4-ethylenedioxypyrrole.

Interestingly, Fradin et al. [23] achieved hydrophobic and oil resistant coatings on nylon and cotton textiles through plasma-induced polymerization of 2-(1H-pyrrol-3-yl) ethanol, 1,3-di (1 H-pyrrol-1-yl) propane, 1,6-di (1 H-pyrrol-1-yl) hexane, and 1,10-di (1H-pyrrol-1-yl) decane monomers. This process produces chemical binding between polymers and fabric fibers, resulting in improved mechanical adhesion.

### 2.2. N-Site Substitution of PPy

Compared with side-substituted pyrrole, N-substituted pyrrole has structural symmetry, giving the polymers a more regular structure and making synthesis more convenient and direct. At the same time, the transition between functional groups is easier. Therefore, the synthesis and performance research of N-substituted PPy derivatives have also received attention. However, the introduction of substituents at the N position, especially when the volume of substituents is large, is generally believed to hinder the polymerization of side positions of pyrrole, reduce the degree of molecular polymerization, and thus shorten the conjugated system. Although the introduction of substituents at the N position may affect the polymerization reaction, the N-position derivatives of PPy have better order and flatness of the skeleton structure, which can bring some new properties [24].

Poly(N-methylpyrrole) (PNMPy) is the simplest N-substituted derivative of PPy [25]. Due to the presence of a methyl group, PNMPy has better mechanical strength, improved anode activity, and lower cost than PPy. At the same time, it is easy to prepare, can be produced in large scale, and has good conductivity [26], biocompatibility, thermal stability [27], and hydrophobicity [28]. It has potential applications in lithium-ion battery materials, antibacterial, corrosion protection, sensors, and other fields.

**Figure 2:**
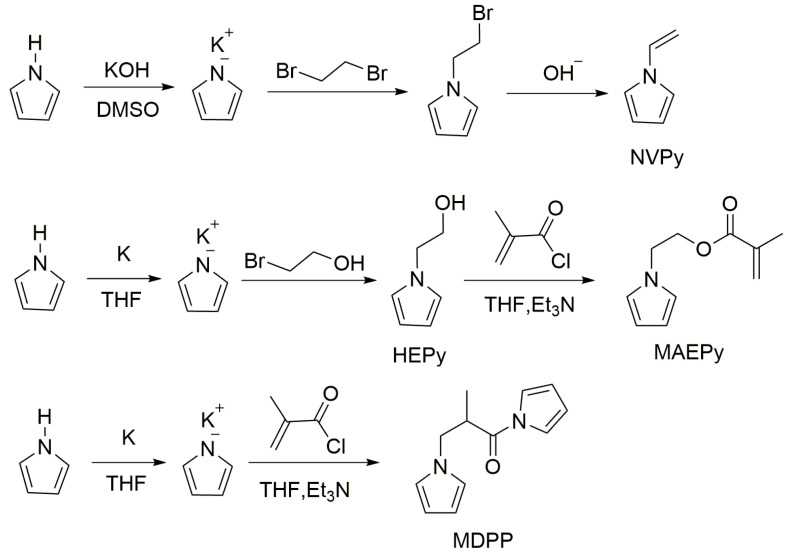
The preparation process of pyrrole derivative monomers by our groups [33,34,35].

In the in-depth study of N-substituted PPy derivatives, researchers found that the solubility of PPy can be improved by introducing some groups through N-substituted [29,30,31,32]. Kijima et al. [29] synthesized three liquid crystalline N-substituted pyrroles using 6-(1-pyrrolyl)hexanol as the reactant through the Mitsunobu reaction. Then, soluble and fusible polymers were obtained through chemical and electrochemical polymerization methods. Our research group prepared the N-vinyl-substituted pyrrole, N-ethyl methacrylate-substituted pyrrole, asymmetric dipyrrole monomer 2-methyl-1,3-di(1-pyrrolyl)-1-acetone (the preparation process is shown in Figure 2), and the corresponding soluble polymers [33,34,35].

**Figure 3:**
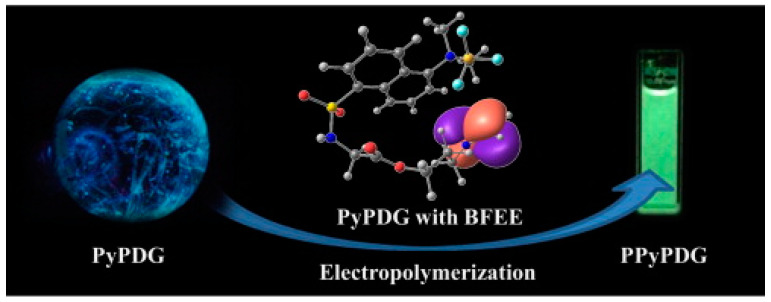
A fluorescent pyrrole derivative bearing a dansyl substituent [36]. Copyright 2014 Electrochimica Acta.

In addition, the PPy derivatives obtained by introducing substituents at the N position also had certain electrochromic properties [36,37,38,39]. Almeida et al. [36] prepared poly(3-(N-pyrryl) propyl dansylglycinate) (PPyPDG) thin films with electrochromic behavior, as shown in Figure 3. Although the color difference in PPyPDG films in the visible region is low (<20%), the color change is observable from greenish yellow (CIE color coordinates x: 0.301, y: 0.173) (neutral state (E = 0.00 V)) to grayish green (CIE color coordinates x: 0.363, y: 0.234) (oxidation state (E = 0.50 V)). In addition, the polymer dissolved in N-methylacetamide solution has fluorescence, showing an emission band at 483 nm, corresponding to light green. The solid-state PyPDG monomer and the soluble polymer PPyPDG also had photoluminescence properties when exposed to ultraviolet light at 366 nm.

In recent years, researchers have prepared a series of structure-controllable, micro-nano N-site-substituted PPy derivatives, including microspheres [40], micro-nano tubes [41,42,43,44], and nanosheets [45]. Our research group [46] used a green method to prepare well-dispersed PNVPY nanoparticles in an aqueous solution and studied the effects of monomer, oxidant, dopant concentration, temperature, UV light intensity, and other conditions on the polymerization rate and product morphology.

### 2.3. Copolymer-Based Pyrrole

Copolymerization is a traditional and effective method for modifying polymer materials, which can make the polymer chain contain two or more repeating unit links, thereby increasing the variety of polymers and expanding the application range of the materials. Generally speaking, the physical and chemical properties of copolymers lie between the homopolymers of the comonomers themselves and are significantly different from these homopolymers and their blend composites. Most conductive polymers or general polymer composites will gradually lose their conductivity due to microphase separation, while various properties of copolymers are relatively stable, because different polymers are connected by covalent bond to form a unified whole [47]. Therefore, a copolymer is a multifunctional material that combines the properties of different comonomers and their respective homopolymers.

**Figure 4:**
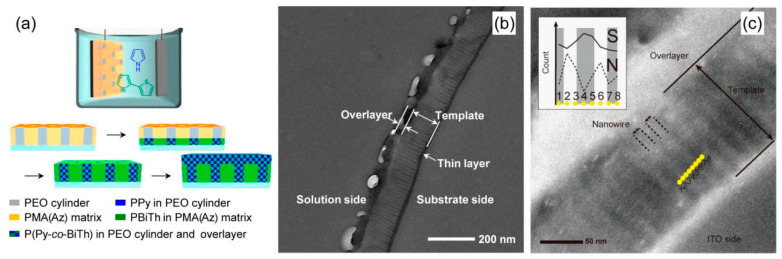
(**a**) Schematic illustration of one-pot electropolymerization using the PEO-b-PMA(Az) template to fabricate laterally integrated conducting polymer nanowires. (**b**) BF-TEM image of an ultrathin section of PEO114-b-PMA(Az)67 template after one-pot electropolymerization of Py and BiTh. (**c**) HAADF-STEM image of the ultrathin section [48]. Copyright 2015 Chemistry of Materials.

For copolymers of PPy, the common comonomers are other conductive polymer monomers, such as aniline, thiophene, etc. [32,48,49,50,51,52,53,54,55,56,57,58]. Furthermore, nanoscale copolymers can also be obtained by copolymerization of pyrrole and other monomers [26,48,56,57,58,59,60,61,62,63]. As can be seen in Figure 4, Komiyama et al. [48] used one-pot electropolymerization to grow pyrrole and 2,2’-dithiophene along PEO cylindrical domains perpendicular to the electrode orientation, resulting in a nanowire array of pyrrole and 2,2’-dithiophene copolymers. Shen et al. [59] used carboxylate azobenzene-4,4′-dicarboxylate disodium salt (ADDS) containing azobenzene group as dopant to controllably synthesize nanostructured polypyrrole derivatives. The results showed that with the guidance of ADDS, the morphology of PPy derivatives changed from nanofibers (nanobelts) to nanosheets and then to granular structure under weak alkaline conditions.

At the same time, it can also endow or improve various properties of pyrrole copolymers, including electrical properties [64,65,66,67], electrochromic properties [50,51], and thermal stability [68,69,70]. Huang et al. [64] proposed a modular organic synthesis method that allowed for the assembly of pyrrole and quinone units into a quinone–pyrrole dimer, and the dimer was modified by changing the substitution mode of the quinone part to fabricate conducting redox polymers (CRPs). On the basis of obtaining CRP materials with adjustable quinone potential, CRP with different quinone potentials was selected as the positive and negative electrode materials to construct an all-organic water-based battery. This type of pyrrole copolymer has achieved satisfactory results in the preparation of sensors, biological antibacterial, energy storage, and microwave absorption applications.

## 3. The Preparation of PPy Derivatives

At present, electrochemical polymerization and chemical oxidation polymerization are mainly used to prepare PPy derivatives. 

### 3.1. Electrochemical Method

By applying an appropriate potential on the electrode, electrochemical polymerization enables the pyrrole derivative monomer in solution to oxidize and polymerize on the electrode surface, and finally forms PPy derivative film on the electrode surface. The polymerization potential can reflect the difficulty of an electrochemical reaction, and the electrode potential of PPy is relatively low in a heterocyclic compound, even lower than polyaniline, so the electrochemical method is one of the main methods to prepare PPy derivatives. The preparation of PPy derivatives by electrochemical polymerization has the following advantages: (1) Simple operation, low energy consumption, and high reproducibility; (2) the size and thickness of the polymer film can be flexibly controlled according to the electrode surface area, polymerization time, current intensity, potential, etc.; and (3) due to the direct deposition of the product obtained by this method on the electrode surface, it can be directly applied to electrode materials of supercapacitors or batteries. However, the electrochemical polymerization method has a low yield, and the reaction process can only be carried out on the electrode, so the area of the membrane is limited by the electrode area, making it impossible to make a large practical conductive film. Therefore, it can only be prepared in small quantities and is difficult to achieve industrialization, which limits its application.

There are three synthesis methods of electrochemical polymerization as follows: the constant voltage method [28,41,48,49], constant current method [38,39,62,65,71], and cyclic voltammetry method [15,18,20,22,27,34,35,37,38,42,64,72]. The constant current method has the advantages of simple operation and equipment, and the easy control of polymerization charge and film thickness. The constant potential method can accurately control the reaction to avoid side reactions, but the polymerization charge and film thickness are not easy to control. Cyclic voltammetry has the same advantages as the potentiostatic method in cyclic potential scanning within a certain potential range. The polymerization reaction was carried out intermittently. The reaction process was mainly carried out in the three-electrode system containing a monomer, electrolyte, and solvent. The polymerization process is mainly influenced by conditions such as types and concentration of monomer [20,27,72], electrode material [38], electrolyte [41], solvent [22,42], doping ions [39,41,42], and polymerization voltage [64]. Each parameter will affect the morphology, thickness, mechanical properties, conductivity, and electrochemical activity of PPy derivative films, thereby affecting the application value of the material. The electrochemical polymerization of PPy derivatives can use metal electrodes such as platinum, gold, stainless steel, as well as graphite and glassy carbon electrodes. Commonly used organic solvents include acetonitrile, benzonitrile, propylene carbonate, etc. The supporting electrolytes used in organic solvents include HClO_4_, NaBF_6_, KPF_6_, TsOH, TsONa, NaNO_3_, H_2_SO_4_, Na_2_SO_4_, etc.

The history of electrochemical synthesis of conductive polymers can be traced back to 1978, when IBM’s Diaz and Kanazawa [73] first used electrochemical methods to prepare PPy films with a conductivity of 100 S/cm in an acetonitrile solution. Subsequently, research on the electrochemical preparation of PPy and its derivatives continued to deepen and was developed in various fields [34,35,36,37,38,39,72,74,75,76,77,78,79,80,81,82]. Wolfart et al. [74] synthesized a copolymer of imidazole and PPy through electrochemical polymerization. As an inhibitor of polymerization, imidazole reduced the total number of active sites polymerized on the electrode surface, resulting in a very different polymer morphology compared with pure PPy, which significantly affected the supercapacitor performance of the modified electrode, increasing the specific capacitance of the material from 122 (pure PPy modified electrode) to 201 Fg^−1^ (imidazole/PPy complex modified electrode), an increase of about 64%.

**Figure 5:**
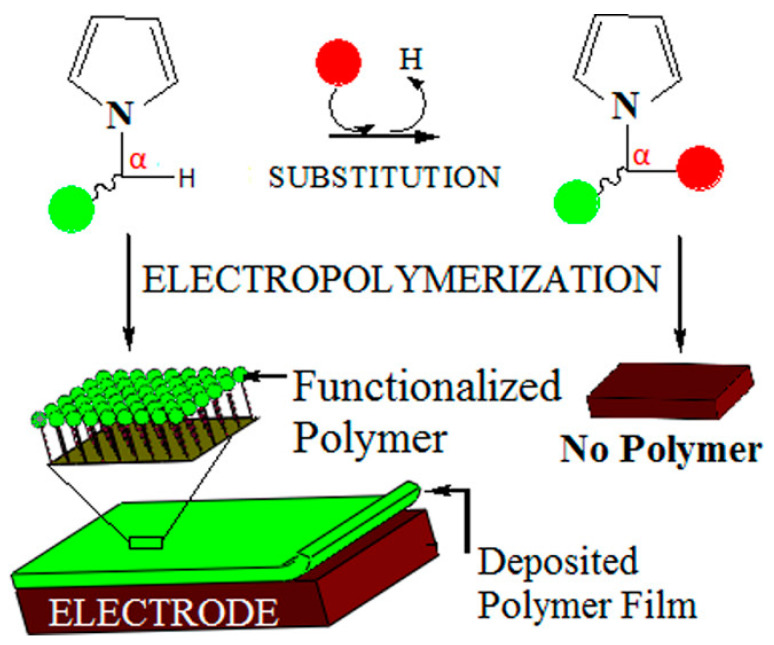
The effect of N-α substitution on the formation of electroactive PPy films [83]. Copyright 2014 Journal of Physical Chemistry C.

In addition, researchers have studied the relationship between structure and electrochemical reaction activity [83,84]. Kumar et al. [83] prepared 21 different N-substituted pyrrole derivatives to study the effect of N-α substitution on the formation of electroactive PPy films. When studying the electropolymerization characteristics of these new N-substituted pyrroles, it was found that, among other factors, the spatial factor caused by α-substitution played the most important role in preventing the electropolymerization of these oxidizable chemicals. Interestingly, even small chemical groups such as CH_3_ in the α-site space are sufficient to prevent the electropolymerization of monomers; the schematic is shown in Figure 5.

### 3.2. Chemical Oxidation Method

**Figure 6:**
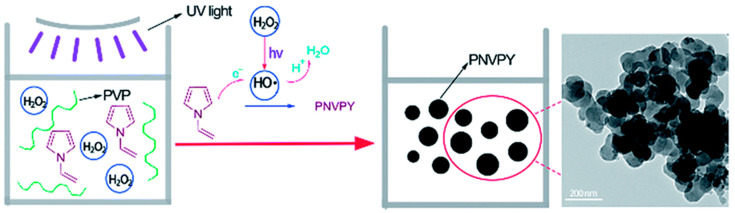
Schematic illustrating the formation of PNVPY nanoparticles [46]. Copyright 2016 RSC Advances.

The chemical oxidation polymerization method utilizes the principle of polymer synthesis to prepare pyrrole polymers by adding suitable oxidants to a certain reaction medium. This method is easy to operate and synthesize, providing various possible synthesis routes with low cost and high yield, making it easy to achieve industrial production. The reaction medium can be a gas phase or liquid phase water, acid solution, and organic solvent (acetonitrile, chloroform, nitromethane, etc.). During the polymerization process, the oxidation and doping of pyrrole derivative monomers occur simultaneously, and the PPy derivative obtained after the reaction is generally in the form of a black powder. The oxidants commonly used to synthesize PPy derivatives include ammonium persulfate, potassium persulfate, hydrogen peroxide, potassium iodate, iron(III) chloride, and other metal salts. Among them, the conductivity of PPy derivatives obtained by using iron(III) chloride as an oxidant was relatively high, and metal salts such as iron(III) chloride could not only be used as oxidant for the synthesis of PPy derivatives, but could also be used as dopant for the conductivity of PPy derivatives, thus it has become the most widely used oxidant for the synthesis of PPy derivatives [85,86,87]. However, when iron(III) chloride is used as an oxidant to synthesize PPy, a large number of metal ions will remain in the product. In addition, the synthesis of PPy derivatives using ammonium persulfate as oxidant is also commonly reported in the literature [88,89]. At present, there are few reports on the synthesis of PPy derivatives using hydrogen peroxide as an oxidant. As hydrogen peroxide is used as an oxidant, the polymerization reaction rate is relatively slow, and the conductivity of the obtained PPy derivatives is slightly low. However, hydrogen peroxide is green and pollution-free, and the reaction by-product is only water. Therefore, it has great potential in the green preparation of PPy. As can be seen in Figure 6, our research group creatively prepared uniformly sized poly(N-vinylpyrrole) nanoparticles using hydrogen peroxide as an oxidant and a UV photocatalytic reaction. The presence of ultraviolet light greatly accelerated the reaction rate and did not introduce other pollutants, providing a new approach for the preparation of conductive polymers using hydrogen peroxide as an oxidant [46].

## 4. Application of Polypyrrole Derivatives

PPy derivatives have many excellent properties and have potential application prospects in various fields such as energy storage, biomedicine, sensors, metal anti-corrosion, electromagnetic shielding, etc.

### 4.1. Energy Storage

Due to their large pseudocapacitance, PPy and its derivatives have been widely studied as energy storage materials in capacitors. Pseudocapacitance refers to the charge stored during the redox process of PPy. Due to the fact that charges are stored throughout the entire volume of PPy, PPy based capacitors can achieve higher specific charge density compared to carbon materials [52,90,91].

**Figure 7:**
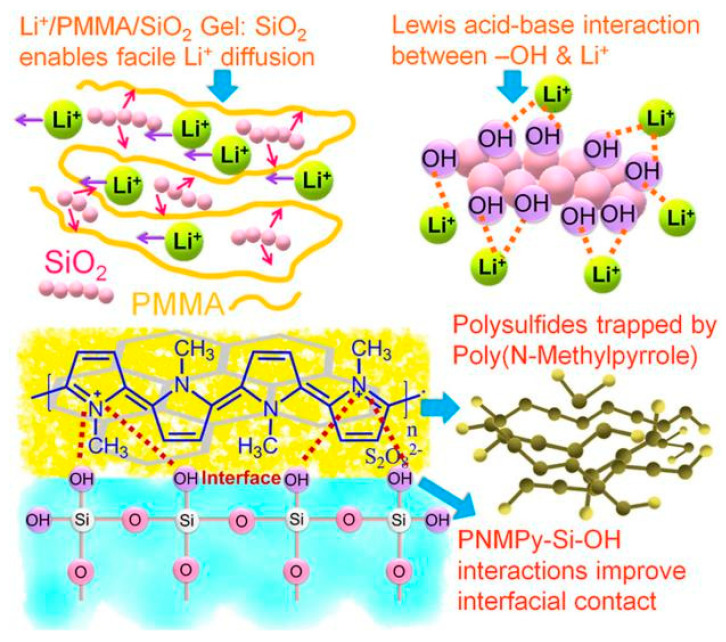
The mechanism of various components in composite materials [93]. Copyright 2020 Electrochimica Acta.

The research of Polypyrrole derivatives in the field of energy storage mainly focuses on lithium batteries [87,92,93,94,95,96,97]. Su et al. [87] prepared a copolymer of 4-(1H-pyrrol-1-yl) phenyl ferrocenecarboxylate (FcPy) and pyrrole (P(FcPy-co-Py)). Due to the resonance doping effect of the side group, the advanced electrochemical properties of the ferrocene part and the loose morphology of the copolymer, the electrode based on P(FcPy-co-Py) exhibits a discharge capacity of 68.1 mAh g-1 and an improved discharge platform. Mukkabra et al. [93] prepared a composite material using PNMPy as the covering layer for S@RGO. The mechanism of various components in composite materials as shown in Figure 7. Due to its high lithium-ion diffusion coefficient of about 10^−6^ cm^2^ s^−1^, it allowed the lithium ions to flow freely between the cathode and the electrolyte during the charge–discharge process. At the same time, it acted as a physical barrier and effectively limited the dissolution and shuttle of Polysulfide thus increasing the capacity retention rate from 13% to 40% after 500 cycles. Yang et al. [97] used a water-based self-assembly to reasonably prepare 5, 10, 15, 20 tetra (4-sulfophenyl) porphyrin (TPPS) crosslinked PPy nanomaterials (PPy–TPPS) through the interaction between -SO_3_^−^ and -NH=^+^. Rigid conjugated TPPS molecules in PPy–TPPS materials bridge linear PPy chains to expand the conjugated system, build multi-path electronic highways, create three-dimensional conductive networks, and greatly enhance the overall conductivity of nanocomposites. In addition, the unique structural features exposed a large number of N-sites, enhanced the chemical anchoring of polysulfide, inhibited the diffusion of polysulfide, and released the shuttle effect.

In addition, the research of Polypyrrole derivatives in a supercapacitor has also been reported [74,89,98,99]. Lacerda et al. [98] synthesized nanohybrids based on carbon nanotubes and EDOT/Py derivative copolymers P(EDOT-co-MPy) and P(EDOT-co-PyMP). Compared with pure capacitance systems (MWCNT) and pure copolymers, the capacitance value of hybrid materials has been improved. Due to the enhanced charge transfer in nanohybrids, the rate capability of the battery has also been improved. Compared to the poor recyclability of conjugated polymers, the device exhibits high performance and excellent cycling stability even after 20,000 cycles.

### 4.2. Biomedicine 

PPy derivatives have become a popular choice for biomedical applications due to their good biocompatibility, easy synthesis, low cost, and rich redox activity.

**Figure 8:**
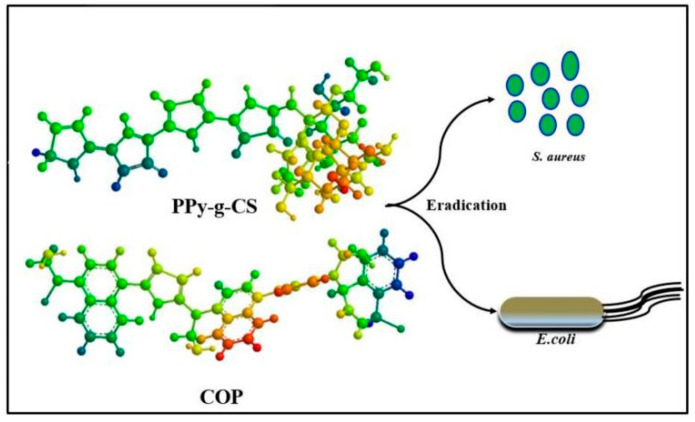
Antibacterial schematic diagram of PPy-g-CS and COP [102]. Copyright 2020 Carbohydrate Polymers.

The research on PPy derivatives in the field of biomedicine mainly focuses on the antibacterial direction [100,101,102,103,104,105]. Ozkazanc et al. [100] investigated the antibacterial properties of Poly(N-methylpyrrole) (PNMPy) and its composite. It was found that the PNMPy and its composite materials showed significant antibacterial effects on Gram-positive *S. aureus* and Gram-negative *E. coli* bacteria due to the hydrophobic effect of the methyl group in the PNMPy structure. The electrostatic interaction between positively charged polymers and negatively charged microbial cell membranes is widely believed to be the cause of cell lysis. Maruthapandi et al. [102] synthesized chitosan-grafted PPy (PPy-g-CS) by a one-step microwave method with carbon dots as the initiator. PPy-g-CS is believed to accumulate on the surface of bacteria, hindering mass transfer and inhibiting their metabolic activity. The antibacterial schematic diagram of PPy-g-CS and COP can be seen in Figure 8. Kumar et al. [103] used a simple one-pot method to synthesize a chitosan (CS) functionalized polyaniline–polypyrrole copolymer (CS/Pani-PPy) to enhance the antibacterial activity of *E. coli* and *E. coli* agglomerates. Research has shown that the synthesized material exhibits excellent antibacterial activity against *E. coli* and *E. coli* agglomerates, and CS/Pani-PPy has observed a decrease of approximately 100% in the activity of these two strains.

In addition, our research group conducted research on the application of two PPy derivatives, PNVPY and PMAEPy, in the field of tissue engineering [106,107]. A preliminary exploration was conducted on the effect of surface conditions on the behavior of nerve cells on conductive electrospun nanofiber membrane scaffolds under electrical stimulation. The study showed that compared to pure electrospun cellulose (EC) scaffolds, more PC12 cells adhered and grew on the PPy derivative-modified fiber membranes, exhibiting a more complete and clear cell morphology. In addition, modified EC membranes can provide more protein adsorption sites due to their unique surface morphology, which is beneficial for cell adhesion and growth. This lays the foundation for the selection of electrical stimulation time and electric field intensity range, as well as the study of the influence of different topological structures, surface morphology, and surface functional groups on cell behavior on composite fiber scaffolds under subsequent electrical stimulation conditions.

### 4.3. Sensors

A sensor is a device that converts temperature, humidity, chemical, and biological information into measurable signals. The output signal of a sensor is usually based on changes in the electrical or optical properties of the material. PPy exhibits reversible changes in conductivity, color, and volume, making it a suitable sensor material. Due to the instability of PPy due to its ability to interact with organic compounds and water in the surrounding environment, PPy sometimes exhibits low sensitivity and selectivity towards the target analyte. In order to improve its sensitivity and selectivity, many attempts have been made to modify PPy [56,57,108,109,110,111,112,113,114,115,116,117,118,119,120,121,122,123,124]. Fabregat et al. [108] prepared poly(N-2-nitrile ethyl pyrrole), poly(methyl pyrrole), and their complexes with gold nanoparticles for low concentration range (10 μM–100 μM) detection of dopamine. The results showed that gold nanoparticles improved the perception ability of both polymers, making both composite polymers highly sensitive to low concentrations of dopamine. Specifically, the composite of poly(N-2-cyanoethylpyrrole) and gold nanoparticles showed a better response and higher efficiency. Ugo et al. [118] deposited poly(1-methyl-3-methylpyrrolidine) on a glassy carbon electrode using an electrochemical method in an acetonitrile solution. By utilizing the anion exchange characteristics of this coated electrode in aqueous solution, HgCI_4_^2−^, a common anionic complex in seawater and other chlorine media, was pre-enriched and detected.

It is worth noting that Jang’s team has performed a lot of research on the application of PPy derivatives in the sensor field and has made some achievements [125,126,127,128]. Their team synthesized carboxylated PPy nanoparticles and nanotubes by chemical oxidation and electrochemical methods and prepared a series of field effect transistor biosensors. The research shows that the sensors made of PPy derivative nanomaterials have high sensitivity, selectivity, and excellent stability for detecting vascular endothelial growth factor, bisphenol A, 17b estradiol, peptide hormone, etc.

**Figure 9:**
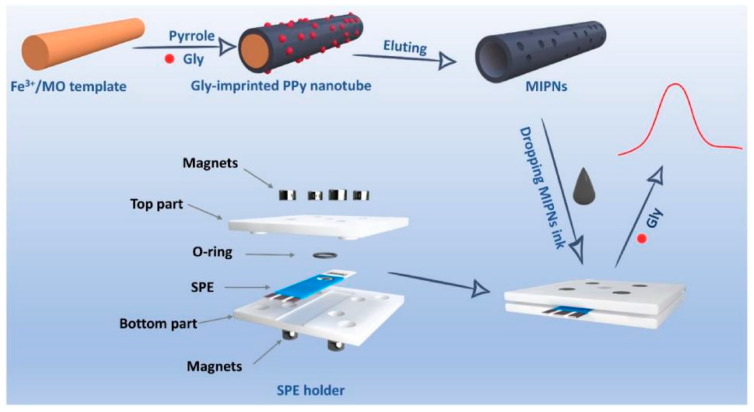
Schematic representation of the designed SPE holder and the principle of MIPNs-based Gly sensor [129]. Copyright 2021 Biosensors and Bioelectronics.

In recent years, molecularly imprinted PPy materials have also been used in the field of sensors [129,130,131]. As shown in Figure 9, Ding et al. [129] prepared molecularly imprinted Polypyrrole nanotubes (MIPNs) by imprinting glyphosate (gly) sites on the surface of PPy nanotubes. Electrochemical sensors based on MIPNs have good stability, high sensitivity, and good reproducibility for gly detection. Table 1 summarizes recent studies on the application of PPy derivatives in sensors.

### 4.4. Corrosion Protection

In the 1980s, DeBerry and Mengoli et al. reported, for the first time, the application of conductive polymers in the field of metal corrosion prevention thus initiating in-depth research on conductive polymer coatings in the field of corrosion prevention [132,133]. Among them, PPy and its derivatives have been widely studied in the field of corrosion protection due to their advantages of easy preparation, good thermal stability, reversible redox potential, low cost, and environmental friendliness [134,135]. Zeybek et al. [134] synthesized poly(N-methylpyrrole)-dodecylsulfate (PNMPy-DS) coatings on stainless steel by potentiodynamic method in oxalic acid solution. Research has shown that PNMPy-DS coating provides effective corrosion protection for stainless steel due to the electrostatic repulsion of corrosive chloride ions by negatively charged large dodecyl sulfate dopants in polymer structures and their delay in entering the metal surface.

Our research group used a green method to prepare various nanostructured PNVPY and studied its anti-corrosion performance on zinc, then compared it with the anti-corrosion performance on zinc of PPy with different structures [136,137].

### 4.5. Others

Intrinsic conductive polymer PPy derivatives have no cytotoxicity, good conductivity and histocompatibility, and are used in electromagnetic shielding field. [138,139] Thi et al. [138] prepared a new composite (P(ANi-co-Py)/RGO@SiO2) consisting of silica nanoparticles coated with poly(aniline–pyrrole) copolymer and reduced graphite oxide by in situ pre-polymerization. Research has shown that the microwave absorption performance of copolymer nanocomposites is significantly improved compared to component homopolymer nanocomposites. The enhancement of microwave absorption is mainly due to interface polarization, where the interface interaction between SiO_2_ particles, rGO sheets, and conductive polymer chains plays a crucial role.

**Figure 10:**
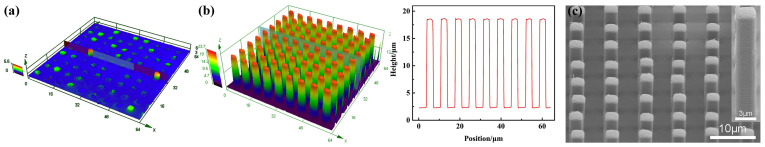
(**a**,**b**) 3D and (**c**) SEM images of the micropatterns for (**a**) PMAEPy and (**b**,**c**) OMADDPy film via EWSF [140]. Copyright 2019 Reactive and Functional Polymers.

In addition, our research group used electric-field-driven micro/nano molding technology to achieve precise and efficient preparation of PPy derivatives with a high aspect ratio of micro/nanostructures. The method of doping the soluble conductive polymers obtained above into the photocurable resin was used to prepare leakage conductive dielectric, and it was theoretically and experimentally confirmed that using leakage conductive dielectric (compared to ideal dielectric) could more quickly prepare its micro/nanostructures with a higher aspect ratio [34,140,141,142] (Figure 10).

## 5. Conclusions and Perspectives

PPy, as a typical conductive polymer, has special optical, electrical, and magnetic properties. Its chemical stability is good, and it is harmless to the environment and organisms. It is an ideal environmentally friendly material and has important significance in energy conservation and sustainable human development. Therefore, it has always received special attention from people. However, PPy has been difficult to process, dissolve, and melt for a long time, which restricts its further research and practical application. The preparation of PPy derivatives endows PPy with new properties such as good solubility, excellent mechanical properties, better thermal stability, excellent photoelectric properties, hydrophobicity, etc. These properties undoubtedly expand the application range of PPy. However, at the same time, maintaining the original conductivity and biocompatibility of PPy is also a problem that cannot be ignored. The selection of suitable substituent groups and comonomers is currently the focus of research. The introduction of substituent groups should ensure that PPy has the required optical, electrical, thermal, magnetic, mechanical, and hydrophobic properties without significantly reducing its conductivity. Comonomers should not only provide PPy with similar copolymerization activity to facilitate the entry of modified monomers into the PPy chain but also enhance the functionality of the resulting copolymer.

Although a series of progress has been made in the application of PPy derivatives, there are still some important challenges. For example, in the field of energy storage applications, supercapacitors composed of PPy derivatives have attracted more and more attention due to their large specific capacitance; however, higher capacitance and better cycling stability are required, which may be improved by optimizing the structure of PPy derivatives or compounding with other materials. For biomedicine applications, the toxicity and non-biodegradability of PPy derivative materials are still important issues to be considered. For the application of polypyrrole derivatives in the field of sensors, the stability of the electrode material in the working environment can only be maintained for tens of days. After that, the responsiveness, sensitivity, and reproducibility are significantly reduced, so the stability needs to be further improved. There are also problems in the application of PPy derivatives in metal corrosion protection. The dispersion of PPy in the film-forming resin is poor, and the detailed anti-corrosion mechanism of PPy derivatives coating is still unclear, all of which need to be further studied. In view of the above problems, the future development of PPy derivatives should focus on the exploitation of new monomers and the improvement of synthesis methods to accurately control the structure and morphology, optimize the performance, and expand the applications. It can be expected that in the coming years, more and more exciting discoveries will be made in these fields.

## Figures and Tables

**Table 1:** A summary of PPy derivatives for sensors.

PPy Derivatives	Analyte	Linear Range	Detection Limit	Ref.
poly(aniline-co-pyrrole) nanocomposites	NH_3_	0.05–20 ppm	0.05 ppm	[56]
poly(aniline-co-pyrrole) nanospheres	NH_3_	10–80 ppm	10 ppm	[57]
PNMPy	dopamine	0.5–2 mM	0.5 mM	[109]
poly[N-(2-cyanoethyl)pyrrole]	dopamine	10–100 μM	6 mM	[111]
ferrocene-conjugated PPy	CO	0–2000 ppm	100 ppm	[113]
PPy-pyrole-2-carboxylic acid copolymer	hCG	100 pg mL^−1^ to 40 ng mL^−1^	2.3 pg mL^−1^	[116]
P(SNS-An-co-EDOT)	O_2_	0.01–5.0 mM	1.9 μM	[117]
poly-[1-methyl-3-(pyrrol-1-ylmethyl)pyridinium]	HgCl_4_^2−^	70–80 nM	0.1 nM	[118]
PPy-COOH nanotube arrays	Cu^2+^	0.1–30 μM	46 nM	[119]
poly(pyrrole-2-carboxylic acid)	Amyloid-beta oligomers	0–10^−4^ pM	10^−4^ pM	[120]
overoxidized PPy-COOH nanowire	Cu^2+^	20–300 nM	20 nM	[122]
Poly-[1-(4-nitronaphthalen-1-yl)-2,5-di(thiophen-2-yl)-1H-pyrrole]	NH_3_	1–300 ppm	1 ppm	[124]
PPy-COOH nanotubes	VEGF	0–4 pm	400 fM	[125]
PPy-COOH nanoparticles	bisphenol A	1–10^4^ fM	1 fM	[126]
PPy-COOH nanotubes	17β-Estradiol	1 fM to 1 nM	1 fM	[127]
PPy-COOH nanoparticles	peptide hormone	4.8 fM to 480 pM	48 fM	[128]
molecularly imprinted PPy nanotubes	glyphosate	2.5–350 ng mL^−1^	1.94 ng mL^−1^	[129]

## Data Availability

Data are contained within the article.
